# Dietary habits among health professionals working in a district hospital in KwaZulu-Natal, South Africa

**DOI:** 10.4102/phcfm.v9i1.1364

**Published:** 2017-06-28

**Authors:** Siyabonga H. Kunene, Nomathemba P. Taukobong

**Affiliations:** 1 Department of Physiotherapy, Faculty of Health Sciences, University of Witwatersrand, South Africa; 2 Department of Physiotherapy, Faculty of Health Sciences, Sefako Makgatho Health Sciences University, South Africa

## Abstract

**Introduction:**

The burden of diseases associated with unhealthy lifestyle behaviours continues to increase in the low- and middle-income countries including South Africa. Among the affected population are the health professionals who are assumed to be knowledgeable about healthy eating.

**Aim:**

This study aimed to determine the dietary habits of health professionals in a public district hospital in KwaZulu-Natal, South Africa.

**Methods:**

A cross-sectional survey was conducted in 2012 among 109 randomly selected health professionals. Each received a questionnaire consisting of mostly closed and few open-ended questions. Its main focus was the dietary and eating habits of the professionals. An ethical clearance was granted by the Medunsa Research and Ethics committee at the University of Limpopo. Permission to conduct the study was sought and obtained from participants as well. Descriptive statistics and frequencies were used to analyse data.

**Results:**

A 100% (109) response rate was achieved. The majority skipped meals especially breakfast with a significant positive correlation between breakfast intake per week and age (*r* = 0.98, *p* = 0.048). The majority consumed a lot of unhealthy foods and carbonated beverages with sugar. Consumption of fruits, vegetables, high fibre and whole grain foods was less common.

**Conclusion:**

The study showed poor eating habits among participants. Urgent health interventions are therefore indicated to highlight the importance of healthy eating habits among the entire population.

## Introduction

Unhealthy eating habits are among the leading critical risk factors for many diseases globally. These factors may lead to conditions such as cardiovascular diseases, diabetes and other types of cancers.^1^ These conditions are noted as contributing highly to the global burden of diseases and need medical attention if health is to be maintained.^1^ This burden of diseases is highest in high-income countries, but is increasing towards a similar profile in low-income countries, contributing towards the rising costs of health care.

The citizens of many African countries including South Africa have poor nutritional status, particularly in terms of energy micronutrient intake.^2^ Among the South African population, there is a complex mix of overnutrition and undernutrition, particularly among women.^3^ Furthermore, the absence of appropriate food, lack of advice on healthy eating habits or cultural food preferences is reportedly increasing the incidence of obesity especially among Black women. The consequence in turn contributes to the growing burden of disease related to lifestyles.

In their capacity as role models for maintaining a healthy lifestyle, health workers play an important role for the health and well-being of the general population. However, studies conducted among medical students and other health workers in many countries, including South Africa, suggest that health workers and students exhibit an unhealthy lifestyle, stemming from physical inactivity and poor eating habits.^4^ In 2015, Kunene et al.^5^ reported that health professionals from a public funded hospital in KwaZulu-Natal presented with low levels of physical activity. This led to a further question of what the dietary habits are among these health professionals. The purpose of this study was therefore to determine the dietary habits of health professionals at the same public funded hospital in KwaZulu-Natal, South Africa.

## Research methods and design

### Study design

A cross-sectional survey that was retrospective in nature was conducted at a single point in time to determine the dietary habits among hospital health professionals in 2012.

### Setting

This study was based in a public funded rural hospital situated in the north-western corner of KwaZulu-Natal province at Estcourt in South Africa. It provides all basic health services with a capacity of accommodating approximately 325 inpatients.

### Study population and sample strategy

Health professionals from the hospital were recruited as participants using a convenience sampling method. Those who participated included doctors, nurses, physiotherapists, occupational therapists, dieticians, radiographers, speech therapists, audiologists and paramedics. All ages, races, genders, nationalities and those in full-time employment for at least six months at the same hospital in the year 2012 were included. The Raosoft statistical tool was used to calculate the sample size of 109 out of 150 participants considering a 95% confidence level.

### Instrument

Literature and other questionnaire tools were used to design a questionnaire for this study. In order to verify this scientific tool, experts were consulted and a pilot study was carried out (15 people). After this approach was conducted, a final version of questionnaire was created. The questionnaire included the following: participants’ demographic profile section, which included gender, age, race, profession and work experience, and dietary habits section, which included mostly closed-ended and few open-ended questions. The questionnaire took each participant approximately 10 min to complete.

### Data collection

Permission from the hospital manager was obtained in writing. An appointment was made with the hospital departments telephonically to collect data. On the day of data collection, the consenting health professionals were given leaflets containing the purpose and objectives of the study and thereafter requested to sign consent forms. Questionnaires were hand distributed to participants at their workstations in the hospital and collected back on the same day after completion.

### Data analysis

Information from the questionnaire was captured on a Microsoft Excel spreadsheet, cleaned and exported to the SPSS statistical program (version 20) for descriptive and inferential analysis. Means, *p* values and Pearson’s correlation were calculated and presented in table form.

### Ethical consideration

Ethical clearance was obtained from the MEDUNSA Research and Ethics Committee (MREC/H/229/2012).

## Results

The study yielded a 100% response rate as all 109 responding health professionals correctly completed and returned the questionnaires.

### Demographic profiles

Most of the participants were Black (82%) and female (83%), with the nursing profession constituting more than half (57%) of the participants, followed by doctors (14%). The remaining professions combined accounted for slightly over a quarter (29%) of the participants. Most (60%) participants were in the ≥ 40-years-old category, and 40% were in the < 40-years-old category. More than half of the participants (54%) had ≤ 5 years of work experience, and just under half (46%) had worked for > 5 years in the same hospital (see Table 1 for full details on the participant’s demographics).

**TABLE 1:** Participants’ demographic profile (*N* = 109).

Demographics	Categories	*N*	(%)
Age	< 40	44	40
	≥ 40	65	60
Race	White	9	8
	Indian	8	7
	Coloured	3	3
	Black	89	82
Work experience	≤ 5 years	59	54
	> 5 years	50	46
Gender	Male	18	17
	Female	91	83
Profession	Doctors	15	14
	Dentists	1	1
	Nurses	62	57
	Physiotherapists	5	4
	Occupational therapists	1	1
	Speech therapists	1	1
	Audiologists	2	2
	Radiographers	4	4
	Dieticians	2	2
	Paramedics	6	5
	Pharmacists	10	9

### Dietary habits

The results in Table 2 show a high percentage of participants that skipped breakfast (51%) compared to lunch (20%) and dinner (11%) meals. A significant positive correlation between breakfast intake per week and age was found to exist among participants (*r* = 0.98, *p* = 0.048). As the age increased, the frequency of breakfast meals taken also increased. A positive correlation between breakfast intake and gender was found, but with no significant difference (*r* = 0.952, *p* = 0.324). No correlation was found between other meals and demographic profile of participants.

**TABLE 2:** The demographics versus meals per week (*N* = 109).

Meals per week	Meals intake per week *N* (%)	Pearson’s correlation and *p*-value			
		Gender	Age	Race	Work experience
Breakfast	Frequently 53 (49) Rarely 55 (51)	*r* = 0.952, *p* = 0.324	*r* = 0.98, *p* = 0.048*	*r* = 0.053, *p* = 0.585	*r* = 0.066, *p* = 0.497
Lunch	Frequently 87 (80) Rarely 22 (20)	*r* = −0.080, *p* = 0.406	*r* = 0.069, *p* = 0.476	*r* = 0.006, *p* = 0.951	*r* = −0.089, *p* = 0.360
Dinner	Frequently 97 (89) Rarely 12 (11)	*r* = −0.033, *p* = 0.520	*r* = −0.063, *p* = 0.517	*r* = −0.003, *p* = 0.784	*r* = −0.034, *p* = 0.727

* significant difference.

Table 3 shows a high percentage of participants who regularly ate dairy foods (74%), meat (91%), sweet foods (60%) and drank carbonated beverages with sugar (55%), coffee (64%) and alcohol (65%). Most participants rarely ate fruits (77%), vegetables (73%) and drank water (68%). The results show a significant correlation that existed between gender and the intake of some food and drink types which included dairy foods (*r* = 1.254, *p* = 0.008), fruits (*r* = 1.169, *p* = 0.078), coffee (*r* = 1.80, *p* = 0.061) and alcohol (*r* = −1.39, *p* = 0.003). As the dairy foods, fruits and coffee intake increased, the scale of correlation moves towards the female gender. As the alcohol intake increased, the scale moves towards the male gender. The results also show another significant correlation between participants’ ages and the intake of tea (*r* = −173, *p* = 0.072) and water (*r* = −1.202, *p* = 0.035). This means, as participants got older, tea and water intake decreased. Another significant correlation existed between work experience and the intake of meat (*r* = −1.225, *p* = 0.019), alcohol (*r* = −1.07, *p* = 0.006) and water (*r* = −1.22, *p* = 0.019). As the work experience increased, the meat, alcohol and water decreased.

**TABLE 3:** Food types and drinks versus demographics (*N* = 109).

Foods	Intake *N* (%)	Pearson’s correlation and *p*-value			
		Gender	Age	Race	Work experience
Dairy food	Frequently 81 (74) Rarely 28 (26)	*r* = 1.254, *p* = 0.008*	*r* = −0.086, *p* = 0.373	*r* = −0.150, *p* = 0.119	*r* = −0.055, *p* = 0.500
Fruits	Frequently 84 (23) Rarely 25 (77)	*r* = 1.169, *p* = 0.078	*r* = −0.044, *p* = 0.652	*r* = −0.082, *p* = 0.394	*r* = 0.001, *p* = 0.993
Vegetables	Frequently 80 (27) Rarely 29 (73)	*r* = −0.089, *p* = 0.358	*r* = −0.046, *p* = 0.638	*r* = −0.039, *p* = 0.685	*r* = 0.048, *p* = 0.623
Meat	Frequently 99 (91) Rarely 10 (9)	*r* = −0.014, *p* = 0.881	*r* = −0.108, *p* = 0.264	*r* = −0.099, *p* = 0.306	*r* = −1.225, *p* = 0.019*
Sweet foods	Frequently 55 (60) Rarely 54 (40)	*r* = −0.035, *p* = 0.719	*r* = −0.142, *p* = 0.141	*r* = 0.058, *p* = 0.373	*r* = −0.128, *p* = 0.186
Carbonated beverage with sugar	Frequently 49 (55) Rarely 60 (45)	*r* = −0.111, *p* = 0.249	*r* = −0.075, *p* = 0.438	*r* = 0.112, *p* = 0.245	*r* = −0.123, *p* = 0.204
Tea	Frequently 34 (31) Rarely 75 (69)	*r* = 0.083, *p* = 0.388	*r* = −1.73, *p* = 0.072	*r* = 0.072, *p* = 0.455	*r* = −0.072, *p* = 0.458
Coffee	Frequently 39 (64) Rarely 70 (36)	*r* = 1.80, *p* = 0.061	*r* = 0.055, *p* = 0.572	*r* = −0.115, *p* = 0.233	*r* = 0.024, *p* = 0.801
Alcohol	Frequently 16 (65) Rarely 93 (35)	*r* = 1.39, *p* = 0.003*	*r* = 0.055, *p* = 0.536	*r* = −0.016, *p* = 0.869	*r* = −1.07, *p* = 0.006*
Fruit juice	Frequently 62 (57) Rarely 47 (43)	*r* = −0.048, *p* = 0.622	*r* = −0.075, *p* = 0.439	*r* = −0.049, *p* = 0.616	*r* = −0.014, *p* = 0.886
Water	Frequently 89 (32) Rarely 20 (68)	*r* = −0.082, *p* = 0.299	*r* = −1.202, *p* = 0.035*	*r* = −1.17, *p* = 0.077	*r* = −1.22, *p* = 0.019*

* significant difference.

Table 4 shows that most participants rarely ate whole grain (53%) and high fibre (61%) foods. Approximately, 50% of participants frequently ate unhealthy snacks, 36% ate salty foods, 37% ate fast foods, 38% ate fatty foods, 49% ate fried foods and 47% ate food with lots of sugar. A significant correlation between age and unhealthy snack intake (*r* = −1.59, *p* = 0.001) and fatty foods (*r* = −1.54, *p* = 0.005) was found. There was no correlation found between other food choices and demographic profile of participants.

**TABLE 4:** Eating habits versus demographics (*N* = 109).

Food choices	Eating habits *N* (%)	Pearson’s correlation and *p*-value		
		Gender	Age	Race
Whole grain food	Frequently 51 (47) Rarely (53)	*r* = −0.103, *p* = 0.287	*r* = −0.045, *p* = 0.642	*r* = −0.237, *p* = 0.433
High fibre food	Frequently 43 (39) Rarely (61)	*r* = 0.066, *p* = 0.499	*r* = −0.017, *p* = 0.858	*r* = −0.057, *p* = 0.555
Unhealthy snacks	Frequently 55 (50) Rarely (49.9)	*r* = 0.155, *p* = 0.678	*r* = −1.59, *p* = 0.001*	*r* = −0.115, *p* = 0.234
Salty food	Frequently 39 (36) Rarely (64)	*r* = −0.048, *p* = 0.619	*r* = 0.039, *p* = 0.684	*r* = 0.144, *p* = 0.136
Fast foods	Frequently 40 (37) Rarely (63)	*r* = 0.165, *p* = 0.564	*r* = 0.032, *p* = 0.742	*r* = 0.073, *p* = 0.450
Fatty food	Frequently 41 (38) Rarely (62)	*r* = −0.128, *p* = 0.185	*r* = −1.54, *p* = 0.005*	*r* = 0.050, *p* = 0.607
Fried food	Frequently 53 (49) Rarely (51)	*r* = −0.003, *p* = 0.974	*r* = 0.108, *p* = 0.263	*r* = 0.087, *p* = 0.368
Sugary food	Frequently 51 (47) Rarely (53)	*r* = 0.026, *p* = 0.791	*r* = 0.050, *p* = 0.608	*r* = −0.089, *p* = 0.355

* significant difference.

### Opinions about food at workplace

Table 5 shows that most participants reported that vegetables (58%) are not sold at their workplace but fruits (56%) are. Most participants reported that fried foods (58%), soft drinks (60%), chips, sweets and cakes (61%) are sold at their workplace. Most of the participants buy from vendors (76%). Few participants buy their food from the outside shops (41%) or eat their own food prepared from home (39%). A high percentage of participants (68%) did not eat at their workplace.

**TABLE 5:** Participants’ opinions about food at their workplace versus demographics (*N* = 109).

Opinions about food at workplace	Responses *N* (%)	Pearson correlation and *P*-Value		
		Gender	Age	Race
Vegetables are sold at work	Agree 46 (42)	*r* = −0.157, *p* =0.103	*r* = 0.031, *p* = 0.748	*r* = −0.063, *p* = 0.518
	Disagree 63 (58)			
Fruits are sold at work	Agree 61 (56)	*r* = −0.161, *p* = 0.233	*r* = −0.047, *p* = 0.628	*r* = −0.047, *p* = 0.624
	Disagree 48 (44)			
Fried foods are sold at work	Agree 63 (58)	*r* = −0.099, *p* = 0.308	*r* = 0.195, *p* = 0.142	*r* = −0.123, *p* = 0.203
	Disagree 46 (42)			
Buy food from vendors	Agree 83 (76)	*r* = 0.024, *p* = 0.804	*r* = 0.071, *p* = 0.461	*r* = −0.060, *p* = 0.533
	Disagree 26 (24)			
Buy food from outside shops	Agree 45 (41)	*r* = 0.029, *p* = 0.763	*r* = −0.011, *p* = 0.906	*r* = −0.026, *p* = 0.789
	Disagree 64 (59)			
Eat prepared food from home	Agree 42 (39)	*r* = −0.131, *p* = 0.174	*r* = 0.085, *p* = 0.378	*r* = 0.039, *p* = 0.691
	Disagree 67 (61)			
Soft drinks are sold at work	Agree 44 (60)	*r* = −0.019, *p* = 0.842	*r* = 0.004, *p* = 0.970	*r* = −0.126, *p* = 0.192
	Disagree 65 (40)			
Chips, sweets and cakes are sold at work	Agree 43 (61)	*r* = 0.038, *p* = 0.698	*r* = 0.163, *p* = 0.323	*r* = −0.155, *p* = 0.108
	Disagree 66 (39)			
Don’t eat at work	Agree 75 (68)	*r* = −0.052, *p* = 0.593	*r* = 0.079, *p* = 0.412	*r* = 0.113, *p* = 0.240
	Disagree 34 (31)			

## Discussion

The results of this study showed that the majority of health professionals do not adhere to healthy eating habits. Most of them skipped meals especially breakfast, and they consumed a lot of unhealthy foods. Other studies also reported a similar problem of unhealthy eating habits among health professionals in various countries including South Africa.^6,7,8,9^ According to Mekary et al.,^10^ most health professionals skipped meals, but male participants skipped meals more as compared to female participants, especially breakfast and lunch meals.

The results of this study showed poor eating habits, which included eating mostly takeaway foods, snacks and fatty foods that are mostly fried and salty. They also drink beverages with a lot of sugar, which include coffee, tea and alcohol. The study has found that these foods participants bought are mostly sold at their workplaces in the tuck shops and from vendors. In a study done in Canada,^11^ physicians were also found not to comply with the acceptable eating standards even though their habits were found to be better than in the general population. It was found that these physicians often do not eat or drink properly, if at all, during working hours.

Skipping meals and eating foods wrongly as mentioned above is not a good health practice as it affects health in many ways. Poor eating habits may result in emotional (irritation and frustration), physical (tiredness and hungriness) and cognitive (difficult concentrating and poor decision-making) challenges.^12^ Lemaire et al. indicated that inadequate workplace nutrition and poor eating habits have a significant negative impact on personal health care, wellness and professional performance.^12^

South Africans are facing a considerable eating health problem that includes a high increase in fast foods (burgers, fried chickens, hotdog, potato chips, milkshakes, etc.), that is, mostly deep fried in oil and salt, consumption as seen in other developing and developed countries today.^9,13^ These eating habit problems among South Africans are mostly as a result of a shift from the basic and natural diet over time towards the Western diet, which is composed of mostly highly unhealthy foods.^14^ Urgent health education programmes and interventions are therefore indicated among health professionals to highlight the importance of healthy eating especially at their workplace where they spend most of their time.

According to WHO in 2015, healthy eating helps protect individuals against malnutrition in all its forms and also protect against noncommunicable diseases (NCDs).^15^ Therefore, it is critical that the energy intake (calories) is balanced with energy expenditure. Evidence suggests that fat should not exceed 30% of total energy intake, and saturated and industrial trans fats should be avoided to prevent overweight and obesity.^16,17^ It is also suggested that individuals should reduce free sugar intake to < 10% of the total energy intake and keep salt intake to < 5 g per day in order to live healthier and avoid disease.^15,17,18^ About 400 g of fruits and vegetables (5 portions) are recommended per day.^17^ Legumes (e.g. lentils and beans), nuts and whole grains (e.g. unprocessed maize, oats, wheat and brown rice) should also be included in the diet of an individual who practises healthy eating.^19^

Healthy eating habits also include enjoying a variety of healthy foods, drinking lots of clean water, eating healthy snacks (e.g. dried fruits) and eating dry beans, split peas, lentils and soya.^19^ It has been suggested that animal product especially red meat and processed meats should be avoided or eaten minimally because of its association with many cancers.^20^ Sugary foods and drinks high in sugar and fried foods should be used sparingly or avoided completely. Alcohol is not required by the body; therefore, it should not be encouraged because of its negative health and social consequences.^19^

## Conclusion

The results of the study showed poor eating habits among the participants. Most participants skipped meals especially breakfast and ate a lot of unhealthy foods. It is interesting to note that the same population also presented with low levels of physical activity according to Kunene and Taukobong in 2015^5^ as indicated in the introduction. Urgent health education programmes and interventions are therefore needed among health professionals to highlight the importance of healthy lifestyle. Hospital management may need to review and come up with strategies to improve eating habits and physical activity. There must be an improvement in the kind of foods and drinks that are provided in their hospital canteens. Failure to adhere to the recommended healthy eating guidelines will continue to increase the burden of NCDs. As role models, health professionals should lead by example in matters of health so that the general population may be encouraged and motivated to adhere to the same health guidelines.

## References

[1] World Health Organization Global strategy on diet, physical activity and health. Geneva: Fifty-seventh World Health Assembly; 2004.

[2] Steyn NP, Whadiah T, Nel J, et al Dietary intake of adult women in South Africa and Nigeria with a focus on the use of spreads [homepage on the Internet]. 2006 [cited 2012 Apr 30]. Available from: www.mrc.ac.za/chronic/kenyareport.pdf

[3] Draper CE, De Villiers A, Lambert EV, et al HealthKick: A nutrition and physical activity intervention for primary schools in low-income settings. BMC Public Health. 2010;10:398–409. https://doi.org/10.1186/1471-2458-10-398 20604914 10.1186/1471-2458-10-398 PMC2910683

[4] Caspersen CJ, Powell KE, Christenson GM Physical activity, exercise, and physical fitness: Definitions and distinctions for health-related research. Public Health Rep. 1985;100:126–131. 3920711 PMC1424733

[5] Kunene SH, Taukobong NP Level of physical activity of health professionals in a district hospital in KwaZulu-Natal, South Africa. S Afr J Physiother. 2015;71:1 https://doi.org/10.4102/sajp.v71i1.234 10.4102/sajp.v71i1.234 PMC6093129 30135872

[6] Ogunjimi LO, Yusuf MM, Olayinka O Prevalence of obesity among Nigeria nurses: The Akwa Ibom state experience. Int NGO J. 2010;5:045–049.

[7] Van den Berg V, Okeyo AP, Dannhauser A, Nel M Body weight, eating practices and nutritional knowledge amongst university nursing students, Eastern Cape, South Africa. Afr J Prim Health Care Fam Med. 2012;4(1):323.

[8] Sun J, Yi H, Liu Z, et al Factors associated with skipping breakfast among inner Mongolia medical students in China. BMC Public Health. 2013;13:42 https://doi.org/10.1186/1471-2458-13-42 23327195 10.1186/1471-2458-13-42 PMC3562148

[9] Feeley AB, Kahn K, Twine R, Norris SA Exploratory survey of informal vendor-sold fast food in South Africa. S Afr J Clin Nutr. 2011;24:199–201. https://doi.org/10.1080/16070658.2011.11734388

[10] Mekary RA, Hu FB, Willett WC, et al The joint association of eating frequency and diet quality with colorectal cancer risk in the health professionals follow-up study. Am J Epidemiol. 2011;175:664–672. https://doi.org/10.1093/aje/kwr363 10.1093/aje/kwr363 PMC3324432 22387430

[11] Frank E, Segura C Health practices of Canadian physicians. Can Fam Physician. 2009;55:810–811.e7. 19675268 PMC2726100

[12] Lemaire JB, Wallace JE, Dinsmore K, Roberts D Food for thought: An exploratory study of how physicians experience poor workplace nutrition. Nutr J. 2011;10:18–25. https://doi.org/10.1186/1475-2891-10-18 21333008 10.1186/1475-2891-10-18 PMC3068081

[13] Van Zyl MK, Steyn NP, Marias ML Characteristics and factors influencing fast food intake of young adult consumers in Johannesburg, South Africa. S Afr J Clin Nutr. 2010;23:124–130. https://doi.org/10.1080/16070658.2010.11734326

[14] Bourne LT, Lambert EV, Steyn K Where does the black population of South Africa stand on the nutrition transition? Public Health Nutr. 2002;5(1A):157–162. https://doi.org/10.1079/PHN2001288 12027279 10.1079/PHN2001288

[15] World Health Organisation Healthy diet [homepage on the Internet] 2015 [cited 2015 Sept 4]. Available from: http://www.who.int/mediacentre/factsheets/fs394/en/

[16] Hooper L, Abdelhamid A, Moore HJ, et al Effect of reducing total fat intake on body weight: Systematic review and meta-analysis of randomised controlled trials and cohort studies. BMJ. 2012;345:e7666 https://doi.org/10.1136/bmj.e7666 23220130 10.1136/bmj.e7666 PMC3516671

[17] World Health Organization Diet, nutrition and the prevention of chronic diseases: Report of a Joint WHO/FAO expert consultation. Geneva: WHO Technical Report Series; 2003; p. 916. 12768890

[18] World Health Organization. Diet [homepage on the Internet] 2012 [cited 2015 May 3]. Available from: http://www.who.int/topics/diet/en/Diet

[19] Department of Health (Republic of South Africa) Guidelines for healthy eating: Information for nutrition educators [homepage on the Internet]. 2013 [cited 2016 Oct 1]. www.nutritionweek.co.za/…/NNW-2013-Nutrition20Educators20Guideline.pdf

[20] International Agency for Research on Cancer Consumption of red meat and processed meat. Lyon: IARC Working Group; 2015; p. 114. 29949327

